# “This has definitely opened the doors”: Provider perceptions of patient experiences with telemedicine for contraception in Illinois

**DOI:** 10.1363/psrh.12207

**Published:** 2022-09-07

**Authors:** Bonnie Song, Angel Boulware, Zarina Jaffer Wong, Iris Huang, Amy K. Whitaker, Lee Hasselbacher, Debra Stulberg

**Affiliations:** ^1^ Department of Obstetrics and Gynecology University of Southern California/LAC+USC Medical Center Los Angeles California USA; ^2^ Department of Comparative Human Development University of Chicago Chicago Illinois USA; ^3^ Department of Family Medicine University of Chicago Chicago Illinois USA; ^4^ Pritzker School of Medicine University of Chicago Chicago Illinois USA; ^5^ Planned Parenthood of Illinois Chicago Illinois USA; ^6^ Center for Interdisciplinary Inquiry and Innovation in Sexual and Reproductive Health (Ci3) University of Chicago Chicago Illinois USA

## Abstract

**Context:**

The COVID‐19 pandemic increased the provision of contraception through telemedicine. This qualitative study describes provider perceptions of how telemedicine provision of contraception has impacted patient care.

**Methods:**

We interviewed 40 obstetrics‐gynecology and family medicine physicians, midwives, nurse practitioners, and support staff providing contraception via telemedicine in practices across Illinois, including Planned Parenthood of Illinois (PPIL) health centers. We analyzed interview content to identify themes around the perceived impact of telemedicine implementation on contraception access, contraceptive counseling, patient privacy, and provision of long‐acting reversible contraception (LARC).

**Results:**

Participants perceived that telemedicine implementation improved care by increasing contraception access, increasing focus on counseling while reducing bias, and allowing easier method switching. Participants thought disparities in telemedicine usage and limitations to the technological interface presented barriers to patient care. Participants' perceptions of how telemedicine implementation impacts patient privacy and LARC provision were mixed. Some participants found telemedicine implementation enhanced privacy, while others felt unable to ensure privacy in a virtual space. Participants found telemedicine modalities useful for counseling patients considering methods of LARC, but they sometimes presented an unnecessary extra step for those sure about receiving one at a practice offering same day insertion.

**Conclusion:**

Providers felt telemedicine provision of contraception positively impacted patient care. Improvements to counseling and easier access to method switching suggest that telemedicine implementation may help reduce contraceptive coercion. Our findings highlight the need to integrate LARC care with telemedicine workflows, improve patient privacy protections, and promote equitable access to all telemedicine modalities.

## INTRODUCTION

The COVID‐19 pandemic motivated the rapid rollout of virtual visits across various healthcare fields, including that of obstetrics‐gynecology (ob‐gyn) and complex family planning. But even prior to the pandemic, digital solutions were emerging as a mode of reproductive healthcare delivery.^1^, ^2^, ^3^ Providing care via telemedicine has shown high levels of patient and provider satisfaction and acceptability for services such as medication abortion and prenatal care.^2^, ^3^, ^4^, ^5^ Studies show that providing care via telemedicine could improve access to medication abortion, and that patients appreciated decreased travel, greater availability of appointment times, and more privacy through telemedicine provision of services compared with in‐person provision.^1^, ^2^, ^6^

In the field of contraception, studies have shown that mobile apps, various online platforms, and virtual visits between patients and providers can be safe and effective means for prescribing contraception, but few have examined the impact of telemedicine provision of contraception on patient care.^7^, ^8^, ^9^, ^10^, ^11^, ^12^, ^13^, ^14^ There is a lack of in‐depth exploration of provider perspectives around how telemedicine provision of services can affect aspects of patient care such as quality of contraceptive counseling and access, which is defined as the degree of fit between characteristics of the patient and the service.^15^ According to Penchansky and Thomas's theory of access, these characteristics are grouped into the dimensions of accommodation, accessibility, availability, affordability and acceptability.^15^ Furthermore, there is a lack of investigation of barriers to contraceptive care after telemedicine implementation, such as disparities in telemedicine uptake or issues with patient privacy, which have been raised in other fields.^12^, ^16^, ^17^, ^18^

When assessing contraception access and counseling, we must also consider the historical context of coercive practices related to reproductive health and how these continue to harm patients, with some groups disproportionately likely to experience coercion.^19^, ^20^, ^21^ Reproductive coercion is defined as any practice that interferes with patients' autonomous decision‐making related to their reproductive health.^22^ Offering alternative modalities to access contraceptive methods and provide counseling through telemedicine could give patients greater control in a way that decreases the likelihood of reproductive coercion compared to a traditional clinic setting.

Understanding provider perspectives of the patient experience allows for a broader synthesis and comparison of experiences across various providers' patient panels. Interviewing providers with experience providing contraceptive services before and after telemedicine implementation can offer valuable insight on how it has impacted patient care. Since providers play an important role in implementation and modification of telemedicine workflows, understanding provider perspectives on improvements and barriers to care with telemedicine implementation will be important for shaping the future of telemedicine provision of contraception.

Findings reported in the quantitative literature neither fully describe what makes telemedicine provision of contraception acceptable nor identify barriers that may prevent some patients from utilizing services for contraception provision.^8^, ^12^, ^23^, ^24^, ^25^ Therefore, the purpose of this study is to gain understanding of provider perspectives around the effect of telemedicine implementation on patient care. We aimed to enhance current understandings by conducting interviews to explore what makes telemedicine provision of contraceptive services a satisfactory and acceptable form of service delivery while identifying barriers that may prevent some patients from receiving care via telemedicine.

## METHODS

### Recruitment

We conducted two rounds of recruitment for qualitative interviews. For the first round of recruitment, we reached out to known professional contacts of research team members who have worked with contraceptive providers on various statewide initiatives through email, contacting clinicians who offered a large volume of contraceptive care in diverse settings. We contacted clinicians in waves using purposive sampling to achieve diversity in geography, practice type (private, academic, or federally qualified health center [FQHC]), and provider type (ob‐gyn and family medicine physicians, physician assistants, and midwives). After our research team secured additional external funding, we conducted a second round of recruitment of staff at Planned Parenthood of Illinois (PPIL). We recruited this participant sample via e‐mail through contacts provided by PPIL collaborators, which included a full list of clinicians and clinical support staff who provided telemedicine services, as well as staff in administration and billing with experience supporting telemedicine operations. All respondents who expressed interest in participation were asked two screener questions determining if they (1) provided or helped provide contraceptive services during the pandemic in Illinois and (2) provided or helped provide care through some telemedicine modality (phone or video) during the pandemic. We considered those who responded “yes” to both screener questions and consented to the study eligible for participation. The University of Chicago Institutional Review Board approved the PPIL section of this study (University of Chicago IRB20‐1387), while the non‐PPIL section received IRB exemption (University of Chicago IRB20‐0981) due to the recruitment of only known professional contacts.

### Data collection

Four researchers trained in qualitative research methods conducted 40 in‐depth interviews of participants and audio‐recorded interviews over Zoom video and phone.^26^ Researchers defaulted to conducting interviews over Zoom video unless participants expressed a preference for phone; five expressed this phone preference. When conducting interviews with Zoom video, we only recorded audio content. Interviews lasted 40–45 min. Researchers conducted interviews of non‐PPIL participants from July 2020 through September 2020. Interviews of PPIL participants took place from January through April 2021. All participants provided oral consent prior to the interview. Participants received a $40 gift card in appreciation of their time after the interview. Researchers converted audio files into written transcripts using the automated transcription function on Zoom, or via a transcription service for phone audio recordings. Researchers then de‐identified and manually verified each transcript. We ended data collection when we believe we had reached thematic saturation.

The data presented in this paper are part of a larger study describing clinicians' experiences providing contraceptive services through telemedicine during the COVID‐19 pandemic. The analysis reported here focuses on the perceived impact of telemedicine implementation on patient care; a separate analysis around operational lessons learned from telemedicine implementation is ongoing. Researchers conducted interviews following a semi‐structured interview guide including 20 stem questions. The questions included in this analysis asked participants about their demographic characteristics, perceived patient response to telemedicine implementation, harms or disruptions to care with telemedicine implementation, and perceived impact of telemedicine implementation on contraception access, LARC (long‐acting reversible contraception, such as intrauterine devices [IUDs] and implants) insertion and removal, efficiency of visits, follow‐up care (including method switching and side effect management), and quality of care. We used the Patient Centered Contraceptive Care (PCCC) framework by Holt and colleagues to guide the creation of the interview guide due to its usefulness as a tool for assessing patient experiences with contraceptive care.^27^ We included interview questions that took into account various contextual factors identified by the PCCC framework (social and economic context, policy and health systems context, and community context) while also evaluating across the continuum of care before, during, and after individuals interact with the healthcare system (outreach, access, quality and follow up support).

### Data analysis

Data analysis followed the principles of thematic analysis, a descriptive strategy facilitating the search for patterns of experience and overarching themes within a qualitative data set.^28^, ^29^ Three researchers read through interview transcripts and developed a codebook using both deductive and inductive techniques. The same three researchers then utilized this codebook to independently code an initial set of transcripts. Through discussion, researchers further refined the definitions of codes and resolved any discrepancies. They repeated this process until they established coding concordance and finalized the codebook. We divided the rest of the transcripts among the same researchers to finish coding using Dedoose software.^30^ Afterwards, 5 researchers developed code summaries highlighting prominent themes to enable in‐depth analysis and synthesis. The researchers extracted notable quotes representative of prominent themes and identified the referenced quotes by the respondent's geography, practice type, and occupation.

## RESULTS

**FIGURE 1:**
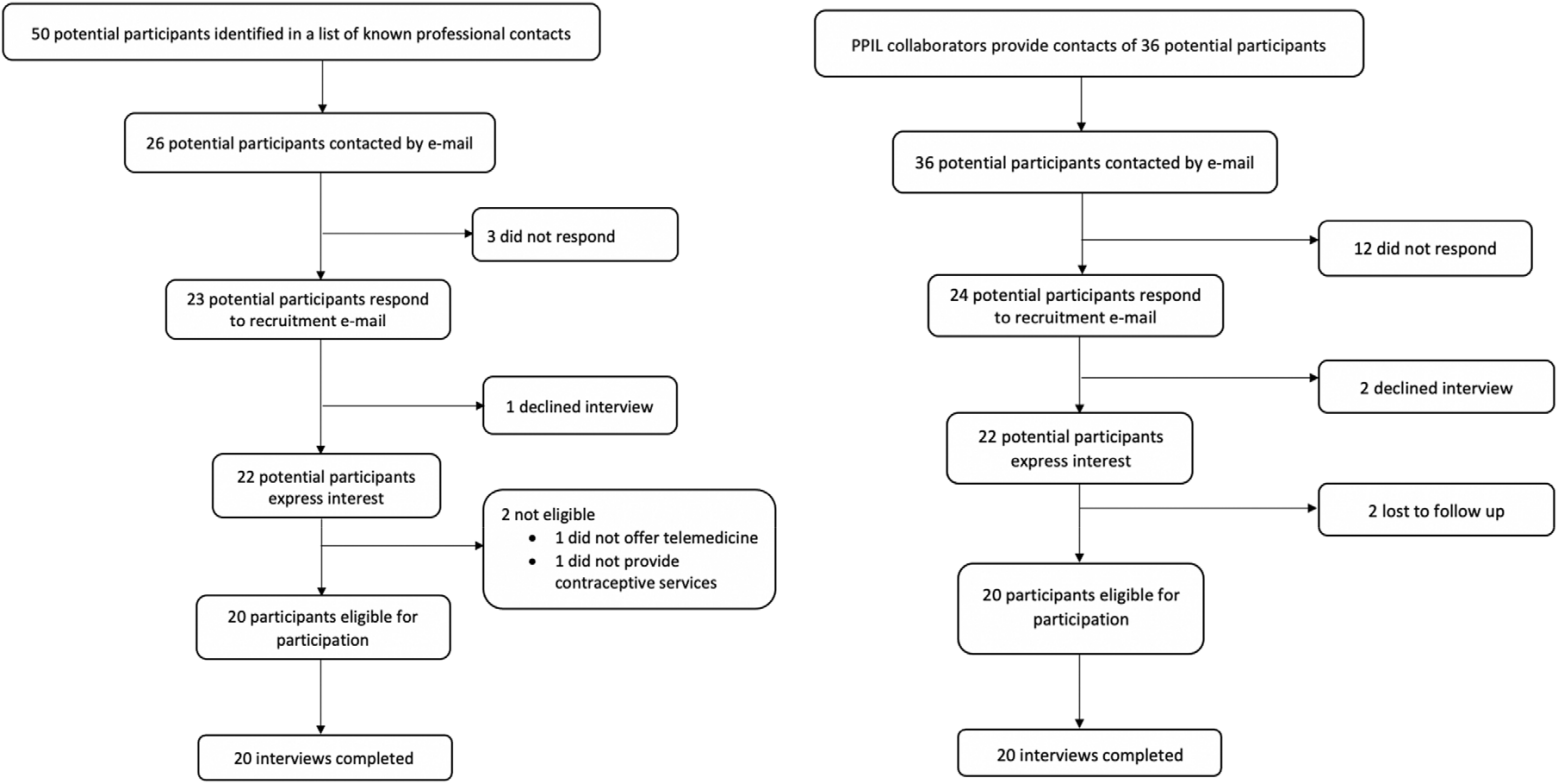
Recruitment process of non‐Planned Parenthood of Illinois (PPIL) and PPIL participants

In the first round of recruitment of contraception providers across diverse practice settings in Illinois, we invited 26 for an interview and 20 completed interviews. In the second round of recruitment of PPIL staff, we invited 36 for interview and 20 completed interviews, for a total of 40 participants in this study (see Table 1). Figure 1 includes details of participant recruitment. Of the 20 non‐PPIL participants, all were clinicians. These clinicians represented 13 different health centers, had 1–34 years in practice, and offered telemedicine provision of contraception for 5–7 months by the time of interview. Of the 20 non‐PPIL participants, all were clinicians. These clinicians represented 13 different health centers, had 1–34 years in practice, and offered telemedicine provision of contraception for 5–7 months by the time of interview. Of the 20 participants from PPIL, the majority were clinicians. Most clinicians worked in Chicago, but a subset also worked in centralized follow‐up sites, a group responsible for responding to phone calls and questions from patients and following up with patients on test results and labs. All participating clinicians in the PPIL sample were nurse practitioners, who make up most of the clinicians at PPIL. PPIL participants represented 11 different PPIL health centers, had 2–11 years of experience in practice, and had offered telemedicine provision of contraception for 13–16 months by the time of interview.

**TABLE 1:** Characteristics of participant sample from various Illinois practices and Planned Parenthood of Illinois (PPIL) health centers (*N* = 40)

Characteristic	Number of participants
Non‐Planned Parenthood of Illinois (PPIL) Sample*	
*By Geography*	
Chicago‐area sites	14
Sites outside Chicago	6
*By Practice Type*	
Community Health Center	11
Academic Health Center	4
Private clinic	5
*By Specialty*	
Ob‐gyn (physicians, midwives)	8
Family medicine/pediatrician (physician, nurse practitioners, physician assistants)	12
*Additional roles*	
Clinic leadership	3
PPIL Sample	
*By Geography*	
Chicago‐area sites	8
Sites outside of Chicago	5
No specific region/statewide	7
*By Position* **	
Clinician (non‐leadership)	11
Clinic support staff	3
Leadership/Administration	6

a All participants in non‐PPIL sample were clinicians.

b All clinicians in PPIL sample were nurse practitioners.

Participants discussed their experiences using telemedicine to provide contraception and shared their perspectives about how telemedicine implementation affects patient care. We organized their responses under three categories: perceived improvements, perceived barriers, and mixed impact on patient care after telemedicine implementation. Perceived improvements included expansion of contraceptive access and improvements to counseling. Perceived barriers to care included disparities in telemedicine access and use and limitations of the technological interface. Mixed impacts included effects on patient privacy and LARC access.

### Perceived improvements to patient care after telemedicine implementation

#### Expansion of contraception access

Participants described an expansion of contraception access with telemedicine implementation. First, all participants in this study reported that telemedicine provision of services did not exist at their practice prior to the pandemic. After telemedicine implementation during the pandemic, participants described an expansion of contraceptive service delivery options, as patients were mostly allowed to choose between an in‐person or telemedicine visit, which increased accommodation by accounting for patient preferences for care. Some participants described increased access with respect to availability. They reported increased availability of appointments, describing that telemedicine appointments tended to be faster visits with less waiting time and were offered sooner than in person appointments. For example, a participant in clinical support at a rural PPIL health center explained, “they were able to get in telehealth within a day, pick up their shot the same day, and stay on their [contraception].”

Furthermore, participants thought telemedicine provision of contraception overcame important barriers related to transportation. A clinician at an urban PPIL health center provided the following example:

I had a patient who has three kids at home, they're all doing homeschooling and she had to renew her birth control […] She doesn't live close by a health center and she just said, ‘You know, I don't have anyone to watch my kids because of everything that's going on. I wouldn't have been able to get my birth control if I didn't have this option,’ so she just, you know, couldn't stop talking about how happy she was that we had that service available.

Some participants noted that they were seeing more patients from rural areas further away from their clinic location. According to one PPIL clinician working from a centralized follow up site, there was “a pretty high uptake of people in more rural areas” who had “limits on transportation.” A family medicine physician in a rural FQHC found telemedicine provision of services was “beneficial” for college students, describing an example of a patient who “was already away at college” so “com[ing] in for an appointment was difficult,” and with telemedicine implementation, “we were able to talk about not liking the birth control pills and really wanting to switch contraception.”

Participants also found that telemedicine provision of services increased accessibility by facilitating follow up care for patients after starting a contraceptive method, such as side effect management and method switching. A rural PPIL health center staff member in clinical support described that “switching a prescription is pretty simple,” but before “you'd have to come into clinic to be reassessed,” and now “through telehealth, it's way faster, way more convenient.” A clinician in an urban PPIL health center also explained:

I've had a few patients lately who booked an appointment because they have had issues or side effects with their method so [telemedicine] is a convenient way for them to follow up with us […] I think they're more likely to follow up if telehealth is available.

#### Improvements in contraceptive counseling

Some participants described improvements in contraceptive counseling with telemedicine implementation, including the potential to mitigate reproductive coercion and bias. Participants thought that providing care via telemedicine allowed for more time to be spent on counseling and education, as they felt less rushed during telemedicine visits and thought they had more time to speak with patients. One ob‐gyn physician at an urban FQHC explained how implementation of virtual visits reduced some of the visit‐related clinic tasks, “so that has actually made me more efficient and I'm able to talk to the patient longer and do better counseling than I even do in the office.” Others thought that patients felt less pressure to decide on a contraceptive method at the same visit. One clinician at an urban PPIL health center described:

If nothing else, [telemedicine] can just be there for an education platform. I think sometimes patients feel like when they actually make an appointment to come into the clinic, they have to have their minds made up about what it is that they want to get. I find that lately I've had a lot more conversation via telehealth about just general education on what method do you feel like is right for me and they don't necessarily feel obligated at that particular visit with accepting a method that they may or may not be prepared to accept in that moment, based on the information that you're given.

Although many participants did not think telemedicine provision of services affected implicit bias in contraceptive counseling, others found that telemedicine provision of services, especially via phone, could help to reduce implicit bias. For example, one midwife at an urban FQHC explained that “a lot of people providing contraceptive care may still approach it with a kind of a paternalistic attitude” and thus “may steer the patient toward the method that they think is better,” with thoughts such as “does she seem to me like a person who would remember to take a pill” based on “that visual component, the physical assessment, how we read the patient.” With phone visits, the participant thought that it could potentially make providers “less biased if we're not visually interpreting” what “we perceive as being evidence that this person is or is not a good candidate for a particular method.” Similarly, a clinical support staff member of an urban PPIL center described that “because when you're using telehealth you're not necessarily using your visual senses, you're actually having to read over the chart, like this is the person's name, this is the person's select pronouns, this is the gender they identify with so people are less likely to assume stuff.”

### Perceived barriers to care after telemedicine implementation

#### Disparities in telemedicine access and use

Several participants commented that telemedicine provision of contraception was more often used among white and insured patient populations. A PPIL clinician working in a centralized follow‐up site described that “we've been looking at our demographics of patients, of telehealth and in person visits […] we're seeing more white and I think insured patients via telehealth,” so “there are some racial disparities of minorities, being able to access telehealth and we're trying to figure out how to increase its access.” More specifically, participants described challenges in access to contraceptive services via telemedicine. First, respondents noted that patients who could not afford high‐speed internet or phones with functional video communication capabilities faced greater barriers to using telemedicine services. One ob‐gyn physician at a rural private practice provided the following example:

She had a phone, but it was broken, so we could only talk for a few minutes at a time, and it was just nuts. We never actually got a chance to complete the whole visit. She never called back to tell me that she had a working phone.

Participants also noted challenges around accommodation. For example, a few participants also noted that patients seemed to prefer phone over video because of “lack of internet access, lack of smartphones” (family medicine physician at a rural academic center) or because “they didn't feel like they looked okay for video” (family medicine physician at urban FQHC). However, providers did not necessarily accommodate patient preferences for phone over video. PPIL participants and some non‐PPIL participants defaulted to video visits over phone visits at their clinic unless patients mentioned not having a phone or computer with working video or if there were technological issues. Their reasons for defaulting to video were perceptions that phone visits would receive lower reimbursement and preferences for seeing their patients. Moreover, respondents described challenges around accommodating patients who did not speak English as their primary language due to the lack of integration of interpreter services with virtual visits compared to in‐person visits, where language lines and interpreters could be more readily dialed or accessed. A PPIL staff member in leadership and administration explained, “we don't have really good integration, or any integration between the telehealth platform and American sign language interpretation or Spanish interpretation.” Furthermore, some providers also reported concerns with inadequate community outreach to accommodate patients unaware of telemedicine provision of services. A family medicine physician at a rural private practice described:

Even just getting it out there, that you can talk about your birth control via telehealth […] some people just fell off the map period. I mean we tried to reach out to them, but a lot of our patients don't have working numbers, or they don't necessarily answer their phone, and we have high no show rates a lot of times… How to just make it known, you could call here?

Lastly, respondents discussed difficulties around acceptability in providing services through telemedicine for those who did not have a private space for conducting the visit. For example, one PPIL clinician at an urban health center described feeling sometimes frustrated with adolescent patients who were with parents or patients who had children around them during the visit, explaining that “we respond to the things as they impact our ability to do our jobs, but sometimes it's easier to have compassion for people when they're sitting in front of you instead of when you're just talking to them through a screen.”

#### Limitations of technological interface

Some participants also discussed limitations of telemedicine implementation, specifically the lack of a physical exam or lab test as barriers to providing high quality patient care. For example, a nurse practitioner at a suburban FQHC described that by providing care via telemedicine, she was no longer able to “couple STI screening with reproductive care” and “routine screening […] has been deferred.” Furthermore, some clinicians reported that navigating the need for blood pressure readings sometimes posed challenges to prescribing estrogen‐based contraceptives via telemedicine. At PPIL health centers, patients who desired estrogen methods were asked to self‐report a blood pressure or go to their nearest pharmacy or clinic for a reading. Several participants not at PPIL health centers stated their clinics gave blood pressure cuffs to at least some of their patients to overcome barriers around providing self‐reported blood pressures. Another concern was around issues with patient engagement. A few participants expressed concerns about patients having distractions at home or not paying attention during virtual visits. They also felt less capable of establishing rapport with patients, as “it's a lot harder to get to know somebody when you're first meeting them through a computer,” according to a clinician at an urban PPIL health center. A midwife at an urban FQHC further elaborated:

I worry particularly with young women and adolescents who have it in their head that all contraceptive methods are the same […] I rely a lot on the rapport that I'm able to establish with them in person […] to make it clear that if they don't like the contraceptive method that they are starting on right now that they can come back, they can call back, we can switch […] I read that the phone call is less personal and they may not, I don't know, establish that same level of trust or believe that I care.

### Mixed impact of telemedicine implementation on patient care

#### Impact on LARC Access

Participants expressed mixed reactions to telemedicine implementation's impact on patient access to and experience of receiving methods of LARC. Participants described the impact of telemedicine implementation on LARC availability and accessibility. Both the pandemic and the implementation of telemedicine meant “we had fewer providers actually in clinic,” so “we were doing less procedure clinics where LARC was available” according to a family medicine physician at a rural academic center. While some thought telemedicine implementation negatively impacted availability of LARC procedures, others thought it positively impacted accessibility of counseling. Because LARC counseling appointments could be done virtually, a family medicine physician at a suburban FQHC thought telemedicine provision “reduces the amount of time [patients] need to spend in the office” so “if they want to go back and talk to their partner” or “talk to their mom,” then it is “easier because we can do the telehealth and that whole piece in preparation first.” A midwife at an urban FQHC described at her practice pre‐pandemic, “it was not uncommon to have one appointment that was the counseling” and then “have [patients] come back on a different day to put it in,” because sometimes, “that has to happen” with “regard to timing of their cycle or most recent unprotected intercourse,” so with telemedicine implementation, patients “don't make two trips when one would suffice.”

However, PPIL participants and many non‐PPIL participants reported that they offered same day LARC insertions. Some of these participants, including one ob‐gyn physician at an urban academic center, mentioned that for “people who know they want LARC devices,” telemedicine visits for LARC could be “frustrating” because “they're not getting it at that first appointment,” thereby negatively impacting accessibility of LARC insertion. Meanwhile, other participants discussed increased accessibility of LARC follow up care via telemedicine. An ob‐gyn physician in a rural private practice explained:

[Telemedicine] actually works. It's probably more efficient than the traditional. For example, where I trained, we routinely after an IUD insertion did a four week IUD string check and now we're not doing that and just talking to the patient over the phone …Typically if they're not having symptoms that are concerning for expulsion […] or malposition then there's no need to actually be there […] We use a lot of Nexplanon and the bleeding profile can be a problem at first. And so just discussing that over the phone, even if it means doing some NSAIDs or additional OCP'S to try and regulate their bleeding […] does not require a physical exam, per se, so telemedicine lends well to that.

#### Impact on patient privacy

Participants expressed mixed perspectives around telemedicine's impact on patient privacy. Some thought patients appreciated receiving contraceptive care in their own home. For that reason, one PPIL clinician in centralized follow‐up described that telemedicine provision of services created “a safe space in some ways that might not have come out in a clinic environment in talking about other social emotional issues that were happening around that time.” Many of the perspectives around telemedicine implementation's impact on privacy were centered around adolescents. Some felt that adolescents appeared more comfortable using telemedicine because, as described by a clinician at an urban PPIL health center, “they're usually seeking confidential services, because they don't want their parents to know” or they “don't want a method that's traceable, like a pack of pills or something that they can actually find,” so telemedicine implementation “could be helpful” because they “could do a telemedicine video while their partner is at work or while they're at school or on their lunch break,” so “there's more flexibility […] as far as a schedule goes if they're trying to hide their actual visit.” Participants thought that providing care in the comfort and privacy of patients' own spaces could protect patients against feelings of stigma in seeking contraception. A primary care physician assistant at an urban FQHC described that telemedicine provision of services “has made things so much better for my transgender population in particular,” and “with contraception, you can have more of a sense of autonomy and control. If you have a door you can close and you can have a conversation without being overheard, then I think you might feel stronger, that your healthcare is your decision and it's going to be more than lip service.” One PPIL participant working in administration highlighted how the privacy allowed by telemedicine provision may be particularly helpful in protecting patients against the stigma they might face in their specific practice setting:

I know because we're Planned Parenthood and the biggest thing that Planned Parenthood is known for is abortion care, that people can be very hesitant to come through our doors in general […] With telehealth, it is […] hopefully a more private setting where the patient can be in a room by themselves […] And then on top of that they're not being seen in public, maybe going to a Planned Parenthood, if they have a partner who is watching them or whatever their situation may be. So, I like to think that this has definitely opened the doors for those patients who, maybe, are scared, because of the stigma that's carried with Planned Parenthood's name, but then it's also a great way for them to see we're not as scary as some people make us out to be.

While many participants appreciated conducting virtual visits in the privacy of a patient's own space, a few also expressed concerns. For example, an ob‐gyn at an academic center expressed that with video visits, “seeing a bit more about the environment that [patients] live and work in, the more cues you pick up about someone probably impacts how you feel about them […] and judgements or biases.”

Many respondents also discussed how maintaining privacy during telemedicine visits could be challenging for adolescents or others with strong privacy needs. One ob‐gyn physician at an urban academic center described, “I can never be totally assured that the patient is […] in a setting that ensures confidentiality.” Another ob‐gyn physician at an urban academic center described her experiences interacting with her patients' parents:

“Lots of times I bring up […] ‘Mom, oh you're used to stepping out when you're at the pediatrician's office,” and that might be a time where they are like, ‘Oh, no, the pediatricians never have me leave’ […] so I think for the younger adolescents, it's harder for me to gauge through telehealth, what the maturity level is, and then talk about the confidentiality stuff.”

Multiple providers mentioned issues with parental interference during telemedicine visits, making it “more difficult for patients to be forthcoming about their needs for contraception,” per a PPIL clinician at an urban health center. One way PPIL staff have worked to address privacy concerns is by creating a chat message with patients at the start of the visit to ensure that they are calling from a safe and confidential location. A ob‐gyn physician at an academic center described her strategy to assessing privacy is by starting the visit with asking her patients, “Do you feel like you're in a place where you're private and you're able to answer sensitive questions,” and “if they say yes, then I know I'm good to go,” but “if they say no, then I need to figure out what my language would be […] around what the hinderance is…could we maybe switch to the chat box and type to each other.”

## DISCUSSION

Providers and staff perceived telemedicine implementation to have an overall positive impact on patient care for contraceptive services. Respondents felt telemedicine provision of services expanded access and improved contraceptive counseling. Following Penchansky and Thomas's theory of access, telemedicine services expanded contraceptive access in multiple dimensions: accommodation, availability, and accessibility.^15^ However, disparities in telemedicine use and limitations of the technological interface also persist as barriers to care. While some patients appreciated receiving care in their own home, challenges with patient privacy arose for those who did not have a private space to conduct the visit. Telemedicine provision of services seems useful for counseling patients unsure about a LARC method or in clinics requiring multiple visits for LARC provision, but it may create an unnecessary extra step for patients who know the method they want at a clinic that offers same day insertion.

As this is a qualitative study intending to capture the perspectives of providers and staff in Illinois, it was not meant to be representative of experiences across the country. The non‐PPIL sample was more of a convenience sample as recruitment was done through known professional contacts, which could limit the transferability of the perspectives represented. However, researchers looked to recruit a diverse sample based on practice type, location, and physician specialty. Additionally, the study sample included participants from the most prolific contraception‐providing centers across Illinois.

This qualitative study is unique in that it evaluates clinician and staff perspectives around the impact of telemedicine provision of contraception on patient care. Just as access to medication abortion expanded after implementation of telemedicine, our findings suggest access to contraceptive services may follow the same trend.^1^, ^2^, ^6^ Two prior studies in New York demonstrated through provider surveys and patient interviews that telemedicine visits are highly effective and satisfactory for providing contraceptive care.^8^, ^11^ The studies showed that patients tended to prefer phone over video visits and had mixed perspectives around impact on privacy, consistent with the perspectives shared in this study.^11^ Our qualitative findings complement those of preexisting studies by highlighting provider and staff perspectives in Illinois, with particular attention to perceived effects on LARC provision, disparities in telemedicine uptake and usage, and improvements in contraceptive counseling. The perceived improvements in counseling and the increased access to follow up care suggest telemedicine implementation could be useful for reducing or disrupting contraceptive coercion, a novel finding to our knowledge. Important to reducing contraceptive coercion is counseling patients about differences among contraceptive methods and ensuring that they are given ample time to make a decision about what is most appropriate for them, while allowing easy access to switching methods.^22^ The improvements in counseling and access to follow up care identified by participants, such as more focus toward counseling and education, the perceived reduction in implicit bias, and easier method switching and side effect management could all contribute to reducing coercion in contraceptive care. Furthermore, the virtual visit itself may also level the power dynamic between physician and patient compared to in person visits where patients enter a physical space clearly demarcated as the “doctor's office” rather than a space of their own.^31^

Our participants' perceptions that telemedicine visits are used more frequently by a predominantly white patient population are consistent with findings from quantitative data in a recent study showing that participants who identified as Black and multiracial were less likely to use telehealth contraceptive services compared to in‐clinic care.^23^ As discussed by our study participants, the disparity may be related to the lack of interpretation services compared to in‐person visits, lack of outreach to patients unaware of telemedicine options, and patients' lack of access to technology or a private space for conducting the visit. Studies have shown that Hispanic, Black, and patients living on low incomes are less likely to have access to regular and adequate internet connection, smartphones and computers.^32^, ^33^

The concerns participants expressed in this study about patients having varying levels of access to and comfort with telemedicine technologies indicate further studies should be conducted to explore patient perspectives around preferences and recommendations to promote equitable access to all telemedicine modalities. There is likely a need to expand access to video visits, but it will also be important to allow patients flexibility and ensure payment parity for both visit types. Illinois enacted legislation in July 2021 requiring phone and video visits to be reimbursed at the same rate as in‐person visits for private insurance plans through 2027; Illinois Medicaid also allows telephonic communications to be reimbursed like video visits.^22^ Ensuring payment parity long term, for plans governed by federal law, and in other states will be important for expanding access and shared decision making in contraceptive care by helping patients receive a contraceptive method in the way most convenient to them. Furthermore, while providers seem to be adequately screening for contraindications and safely providing birth control methods to patients,^10^ respondents in this study discussed challenges in capturing blood pressure readings when prescribing estrogen‐based contraceptives. Thus, more clinics may benefit from providing blood pressure cuffs to patients. Our findings also suggest that further interventions are needed to improve patient privacy, such as chat functions to offer safety check‐ins. Lastly, our findings around the perceived impact on LARC suggest that clinics may benefit from established protocols that better integrate telemedicine workflows with LARC, which may vary depending on a patient's readiness for LARC and whether the practice offers same day insertion.

## CONCLUSIONS

Virtual healthcare visits have become an important way to deliver essential healthcare services such as contraception while allowing patients the access and flexibility to receive care from a space of their choosing. Telemedicine provision of contraception is not without its challenges. Many of the concerns that contraception providers expressed, such as concern for telemedicine access or patient discomfort with being on video are similar to the concerns related to telemedicine provision of services faced by clinicians from other disciplines.^16^, ^17^, ^18^ However, given the stigma associated with contraceptive services, many of the concerns expressed are also around issues that have long been centralized in the field of family planning, such as prioritizing patient privacy, valuing patient autonomy and preference in clinical decisions, and working on challenging implicit bias. If able to address these issues in telemedicine provision of contraception, this service could be a model for other fields as healthcare adapts to new circumstances.
